#  Transduction of the *Streptococcus pyogenes* bacteriophage Φm46.1, carrying resistance genes *mef*(A) and *tet*(O), to other *Streptococcus* species

**DOI:** 10.3389/fmicb.2014.00746

**Published:** 2015-01-08

**Authors:** Eleonora Giovanetti, Andrea Brenciani, Gianluca Morroni, Erika Tiberi, Sonia Pasquaroli, Marina Mingoia, Pietro E. Varaldo

**Affiliations:** ^1^Unit of Microbiology, Department of Life and Environmental Sciences, Polytechnic University of MarcheAncona, Italy; ^2^Unit of Microbiology, Department of Biomedical Sciences and Public Health, Polytechnic University of Marche Medical SchoolAncona, Italy

**Keywords:** *Streptococcus* species, Φm46.1, bacteriophages, mef(A), tet(O), transductionchromosomal, integration, fitness cost

## Abstract

Φm46.1 – *Streptococcus pyogenes* bacteriophage carrying *mef*(A) and *tet*(O), respectively, encoding resistance to macrolides (M phenotype) and tetracycline – is widespread in *S. pyogenes* but has not been reported outside this species. Φm46.1 is transferable *in vitro* among *S. pyogenes* isolates, but no information is available about its transferability to other *Streptococcus* species. We thus investigated Φm46.1 for its ability to be transduced *in vitro* to recipients of different *Streptococcus* species. Transductants were obtained from recipients of *Streptococcus agalactiae*, *Streptococcus gordonii*, and *Streptococcus suis*. Retransfer was always achieved, and from *S. suis* to *S. pyogenes* occurred at a much greater frequency than in the opposite direction. In transductants Φm46.1 retained its functional properties, such as inducibility with mitomycin C, presence both as a prophage and as a free circular form, and transferability. The transductants shared the same Φm46.1 chromosomal integration site as the donor, at the 3′ end of a conserved RNA uracil methyltransferase (*rum*) gene, which is an integration hotspot for a variety of genetic elements. No transfer occurred to recipients of *Streptococcus pneumoniae*, *Streptococcus oralis*, and *Streptococcus salivarius*, even though *rum*-like genes were also detected in the sequenced genomes of these species. A largely overlapping 18-bp critical sequence, where the site-specific recombination process presumably takes place, was identified in the *rum* genes of all recipients, including those of the species yielding no transductants. Growth assays to evaluate the fitness cost of Φm46.1 acquisition disclosed a negligible impact on *S. pyogenes*, *S. agalactiae*, and *S. gordonii* transductants and a noticeable fitness advantage in *S. suis*. The *S. suis* transductant also displayed marked overexpression of the autolysin-encoding gene *atl*.

## INTRODUCTION

Many bacterial genomes deposited in public databases contain phage DNA integrated into the bacterial chromosome, at times as multiple prophages. Prophages are not passive genetic cargo of the bacterial chromosome but are likely to be active players in cell physiology, since phage DNA is a vector, as other mobile genetic elements, for lateral gene transfer between bacteria (Canchaya et al., 2003).

A neat test case for the role of prophages is *Streptococcus pyogenes* (Banks et al., 2002). About 90% of the isolates of this species are lysogenic, due to complete or partial prophages integrated into the host chromosome that sometimes contribute up to 12% of the total genome (Canchaya et al., 2003; Ferretti et al., 2004). Transformation appears to play no or only a minor role in lateral DNA transfer in *S. pyogenes*, conferring on phages a special role in this process. It has been suggested that in this species competence and transformation may have been lost due to the growing role assumed by bacteriophages in population diversity (Ferretti et al., 2004). These *S. pyogenes* phages or phage-like elements have long been known to encode many virulence factors, but more recently they have also been shown to carry antibiotic resistance genes. In particular, this applies to the macrolide eﬄux resistance gene *mef* (A) (Clancy et al., 1996), which is typically associated with a low-level resistance pattern involving, among macrolide-lincosamide-streptogramin B antibiotics, only 14- and 15-membered macrolides (M phenotype; Sutcliffe et al., 1996b). *mef* (A) is carried by Tn*1207.1*, a defective transposon originally detected in *Streptococcus pneumoniae* (Santagati et al., 2000). In *S. pyogenes*, Tn*1207.1* is not found as such, but as part of larger composite elements that have all been shown to be chimeric, i.e., resulting from insertion of a transposon (identical or related to Tn*1207.1*) into a prophage (Banks et al., 2003; Giovanetti et al., 2005).

The *mef* (A)-carrying phage varies depending on whether the strain is resistant only to macrolides or also to tetracycline. When M-phenotype isolates of *S. pyogenes* are tetracycline susceptible, the bacteriophages involved are Φ1207.3 (formerly Tn*1207.3*, 52,491 bp, accession no. AY657002) (Santagati et al., 2003; Giovanetti et al., 2005; Iannelli et al., 2014) or Φ10394.4 (58,761 bp, accession no. AY445042; Banks et al., 2003, 2004), which are closely related and are integrated into the same chromosomal gene (*comEC*, encoding a putative competence protein; Santagati et al., 2003; Banks et al., 2003; Brenciani et al., 2004). The only difference is that, in Φ1207.3, Tn*1207.1* is the left end of the element, whereas Φ10394.4 presents an additional left-hand region of ∼6 kb. When M-phenotype isolates of *S. pyogenes* are coresistant to tetracycline – a condition that in Italy is more common than macrolide resistance alone (Giovanetti et al., 1999; Brenciani et al., 2004; D’Ercole et al., 2005) – tetracycline resistance is mediated by the *tet*(O) determinant (Giovanetti et al., 2003), linked to *mef* (A) in a phage variety whose extensively investigated representative is Φm46.1 (55,172 bp, accession no. FM864213; Giovanetti et al., 2005; Varaldo et al., 2009; Brenciani et al., 2010). Compared to Φ1207.3/Φ10394.4, Φm46.1 has a different integration site, at the 3 ° end of a chromosomal gene (*rum*) encoding an RNA uracil methyltransferase (Brenciani et al., 2010). Electron microscopic analysis following induction with mitomycin C has revealed phage particles with the typical icosahedral head and tail morphology of *Siphoviridae* in both Φ10394.4 (Banks et al., 2003) and Φm46.1 (Brenciani et al., 2010).

Φ1207.3 and Φ10394.4 have also been detected in *Streptococcus* species other than *S. pyogenes*: the former in *Streptococcus agalactiae* (Marimón et al., 2005) and the latter in viridans group isolates of *Streptococcus gordonii* and *Streptococcus salivarius* (Brenciani et al., 2014). Conversely, Φm46.1 has never been reported outside *S. pyogenes*. Moreover, in *in vitro* transfer experiments Φ1207.3 was transferred to other *Streptococcus* species (Santagati et al., 2003), whereas such experiments have never been performed with Φm46.1. In early conjugation assays using *S. pyogenes* donors whose *tet*(O)–*mef* (A) elements had not yet been realized to be phages, *mef* (A) and *tet*(O) were co-transferred to a *S. pyogenes* but not to an *Enterococcus faecalis* recipient (Giovanetti et al., 2003). More recently lysogenic transfer of Φm46.1 has been reported among *S. pyogenes* isolates (Di Luca et al., 2010).

In this study, we investigated the ability of Φm46.1 to be transduced to recipients of *Streptococcus* species other than *S. pyogenes*. Φm46.1 was transferred to some species but not to others. The chromosomal integration site of Φm46.1 in the transductants corresponded to the one originally detected in *S. pyogenes*. Investigation of the fitness cost associated with Φm46.1 acquisition disclosed that it varied with the species and that a significant fitness advantage was conferred on the *Streptococcus suis* transductant.

## MATERIALS AND METHODS

### BACTERIAL STRAIN

The strain harboring Φm46.1 was the same (*S. pyogenes* m46, an ST39, *emm* type 4 throat clinical isolate) where the *mef* (A)–*tet*(O) combination and linkage were initially detected (Giovanetti et al., 2003), and from which Φm46.1 was subsequently characterized and sequenced (Brenciani et al., 2010). Phenotypically, the strain is coresistant to erythromycin (MIC, 16 μg/ml; M phenotype) and tetracycline (MIC, 64 μg/ml).

### ANTIBIOTICS AND SUSCEPTIBILITY TESTS

Erythromycin and tetracycline were purchased from Sigma Chemical Co. (St. Louis, MO, USA). MICs were determined by a standard broth microdilution method, using *S. pneumoniae* ATCC 49619 for quality control.

### LYSOGENIC TRANSFER AND ANALYSIS OF TRANSDUCTANTS

Transfer experiments were performed as described elsewhere (Giovanetti et al., 2002). *S. pyogenes* m46 was used as the donor. Rifampin- and fusidic acid-resistant (RF) derivatives of erythromycin- and tetracycline-susceptible strains of different *Streptococcus* species were used as recipients: *S. pneumoniae* R6RF (Cochetti et al., 2005); *S. agalactiae* 1357RF (Palmieri et al., 2012); *S. gordonii* 1435RF (Mingoia et al., 2014); *Streptococcus oralis* 1235RF (Mingoia et al., 2014); *Streptococcus suis* v36RF (Palmieri et al., 2012); and *S. salivarius* 1555RF, an RF derivative obtained for this study from a recently investigated strain (Brenciani et al., 2014). Retransfer experiments were performed using *S. pyogenes* 12RF-SN, a streptomycin- and nalidixic acid-resistant derivative of our recipient 12RF (Giovanetti et al., 2003), as the recipient. Transductants were selected on plates containing erythromycin (1 μg/ml) plus rifampin (10 μg/ml) and fusidic acid (10 μg/ml), or plus streptomycin (500 μg/ml) and nalidixic acid (10 μg/ml) in retransfer assays. Putative transductants were tested for *mef* (A) and *tet*(O) by polymerase chain reaction (PCR) and for erythromycin and tetracycline MICs; the presence of Φm46.1 was checked by PCR mapping in five randomly selected transductants of each species. Transduction frequency was expressed as the number of transductants per recipient. Mating experiments were done at least three times.

### PCR EXPERIMENTS

The primer pairs used in PCR experiments are listed in **Table 1**. DNA preparation and amplification and electrophoresis of PCR products were carried out by established procedures and following recommended conditions for the use of individual primer pairs. The Ex Taq system (TaKaRa Bio, Shiga, Japan) was used when expected PCR products exceeded 3 kb in size.

**Table 1:** Oligonucleotide primer pairs used.

	Primer			
Procedure Gene	Designation	Sequence (5′–3′)	Reference or source	Product size (bp)
**Resistance genes**
*mef*(A)	MEFA1	AGTATCATTAATCACTAGTGC	Sutcliffe et al. (1996a)	
*mef*(A)	MEFA2	TTCTTCTGGTACTAAAAGTGG	Sutcliffe et al. (1996a)	348
*tet*(O)	TETO1	AACTTAGGCATTCTGGCTCAC	Olsvik et al. (1995)	
*tet*(O)	TETO2	TCCCACTGTTCCATATCGTCA	Olsvik et al. (1995)	519
**Φ m46.1 junctions (*****S. agalactiae*** **chromosome)**^**a**^
SAG0633	RUMSa-for	GTGTCTGCCTTTCCTTCTGTTGT	This study	
*mef*(A)	MEFA2	TTCTTCTGGTACTAAAAGTGG	Sutcliffe et al. (1996a)	3,328
*tet*(O)	TETO1	AACTTAGGCATTCTGGCTCAC	Olsvik et al. (1995)	
SAG0635	PHOSa-rev	CTAACAGTAATCGGCTTCTT	This study	5,994
**Φ m46.1 junctions (*S. gordonii* chromosome)^**a**^**
SGO-1364	RUMSg-for	GCGAGTTCTCAAAGTCAATAAAA	This study	
*mef*(A)	MEFA2	TTCTTCTGGTACTAAAAGTGG	Sutcliffe et al. (1996a)	3,887
*tet*(O)	TETO1	AACTTAGGCATTCTGGCTCAC	Olsvik et al. (1995)	
SGO-1361	ADPSg-rev	CTCAGCAACAGCGCAGGTCA	This study	7,429
**Φ m46.1 junctions (*S. suis* chromosome)^**a**^**
SSUD9_0757	rum-F	GCATCTCACTTATCCAGCCC	Palmieri et al. (2011a)	
*mef*(A)	MEFA2	TTCTTCTGGTACTAAAAGTGG	Sutcliffe et al. (1996a)	3,764
*tet*(O)	TETO1	AACTTAGGCATTCTGGCTCAC	Olsvik et al. (1995)	
SSUD9_0755	glf-R	CCTCGTTTCCAGGTCTTCG	Palmieri et al. (2011a)	7,223
**Chromosomal empty target^**b**^**
SAG0633	RUMSa-for	GTGTCTGCCTTTCCTTCTGTTGT	This study	
SAG0635	PHOSa-rev	CTAACAGTAATCGGCTTCTT	This study	1,298
SGO-1364	RUMSg-for	GCGAGTTCTCAAAGTCAATAAAA	This study	
SGO-1361	ADPSg-R	CTCAGCAACAGCGCAGGTCA	This study	3,326
SSUD9_0757	rum-F	GCATCTCACTTATCCAGCCC	Palmieri et al. (2011a)	
SSUD9_0755	glf-R	CCTCGTTTCCAGGTCTTCG	Palmieri et al. (2011a)	2,597
**Φ m46.1 circular form**
*orf1*	ORF1-rev	TAATAAGTGAGAGCAAGTTG	This study	
*orf63*	ORF63-for	CAGATGGATGGTGTTTCAG	This study	788
**Chromosomal gene used in phage induction experiments (*S. pyogenes*)**
*speB*	SPEB1	ACCGTGTTATTGTCTATTACC	Banks et al. (2003)	
*speB*	SPEB2	TGCCTACAACAGCACTTTGG	Banks et al. (2003)	1,300
**Chromosomal gene used in phage induction experiments (*S. agalactiae*)**
*tkt*	tkt-fw	CCAGGCTTTGATTTAGTTGA	Jolley and Maiden (2010)	
*tkt*	tkt-rw	AATAGCTTGTTGGCTTGAAA	Jolley and Maiden (2010)	859
**Chromosomal gene used in phage induction experiments (*S. gordonii* and *S. suis*)**
*recA*	recA-up	TATGATGAGTCAGGCCATG	King et al. (2002)	
*recA*	recA-dn	CGCTTAGCATTTTCAGAACC	King et al. (2002)	421
**Quantitative real-time reverse transcription-PCR**
*atl*	atl-for	TAACAGGTGCGGGTGGAACA	This study	
*atl*	atl-rev	ATCTGACTGACGAGTGGCTT	This study	206
rDNA16s	P891F	TGGAGCATGTGGTTTAATTCGA	Warwick et al. (2004)	
rDNA16s	P1033R	TGCGGGACTTAACCCAACA	Warwick et al. (2004)	159

*^a^First primer pair, left junction; second primer pair, right junction.*

*^b^First primer pair, *S. agalactiae*; second primer pair, *S. gordonii*; third primer pair, *S. suis*.*

### DNA SEQUENCING AND SEQUENCE ANALYSIS

All PCR products used for sequence analysis were purified using Montage PCR filter units (Millipore Corporation, Bedford, MA, USA). Amplicons were sequenced (bidirectionally or by primer walking) using ABI Prism (Perkin-Elmer Applied Biosystems, Foster City, CA, USA) with dye-labeled terminators. Sequences were analyzed using the Sequence Navigator software package (Perkin-Elmer Applied Biosystems). Sequence similarity and conserved domain searches were carried out using tools (BLAST and CDART) available online at the National Center for Biotechnology Information of the National Library of Medicine (Bethesda, MD, USA; ).

### INDUCTION OF Φm46.1 WITH MITOMYCIN C

For phage induction, one transductant of each species was treated with 0.2 μg/ml mitomycin C (Sigma) for 4 h at 37°C. *S. pyogenes* m46 was used as a control; phage DNA was extracted and purified as described previously (Brenciani et al., 2010). Induction was monitored by PCR using primer pairs targeting *mef* (A) and *tet*(O). Chromosomal genes (*speB* for *S. pyogenes*, *tkt* for *S. agalactiae*, and *recA* for *S. gordonii* and *S. suis*) were monitored as negative controls using specific PCR primers (**Table 1**) to confirm that there was no contaminating chromosomal DNA in the phage DNA preparations.

### FITNESS ASSESSMENT

The biological cost of Φm46.1 acquisition was investigated by growth assays, fitness differences being disclosed by exponential growth rates measured in resistant transductants and susceptible recipients. Competitive growth assays could not be used due to transduction events occurring during co-culture of recipients and transductants. One transductant and the recipient of each species [from previous experiments in the case of *S. pyogenes* (Giovanetti et al., 2003)] were grown overnight in brain heart infusion broth (BHI; Difco Laboratories, Detroit, MI, USA) at 37°C in 5% CO_2_. Cultures were diluted to an optical density (OD) of 0.1 ± 0.05 at 690 nm and then diluted 1:100 in BHI. From each dilution, a 150-μl aliquot was transferred to a well of a microtiter plate. Growth was monitored at 37°C for 24 h using Multiscan Ascent (Thermo Scientific, Waltham, MA, USA); OD_690_ measurements were taken every hour. Experiments were repeated three times.

### RNA ISOLATION AND QUANTITATIVE REAL-TIME REVERSE TRANSCRIPTION-PCR (qRT-PCR)

Cultures were grown overnight in BHI at 37°C and then diluted 100-fold in fresh BHI. Subcultures were collected at the logarithmic phase (OD_690_ value of 0.8), and total RNA was isolated with the GenElute total RNA purification kit (Sigma) according to the manufacturer’s instructions. Total cDNA was obtained by the QuantiTect Reserve Transcription kit (Qiagen, Hilden, Germany) according to the manufacturer’s instructions. The copy number of a specific cDNA was calculated using the Rotor-Gene Q MDx instrument and the Rotor-Gene SYBR Green PCR kit (Qiagen). The 16S rDNA housekeeping gene was analyzed as an internal control. The primers used for qRT-PCR assays are reported in **Table 1**.

## RESULTS

### LYSOGENIC TRANSFER AND ANALYSIS OF TRANSDUCTANTS

Transfer of macrolide and tetracycline coresistance from *S. pyogenes* m46, formerly described to a *S. pyogenes* recipient (Giovanetti et al., 2003), was obtained in the present study to recipients of *S. agalactiae*, *S. gordonii*, and *S. suis*, but not of *S. pneumoniae*, *S. oralis*, and *S. salivarius* (**Table 2**). In retransfer experiments using transductants as donors, including a previous *S. pyogenes* transductant (Giovanetti et al., 2003), erythromycin and tetracycline coresistance was consistently retransferred to *S. pyogenes* (**Table 2**). Whereas transduction frequencies were comparable (or slightly lower) in retransfer *vs.* transfer assays with *S. pyogenes*, *S. agalactiae,* and *S. gordonii*, retransfer from *S. suis* to *S. pyogenes* occurred at a much greater frequency (over 10^4^ times) than transfer from *S. pyogenes* to *S. suis*.

**Table 2:** Lysogenic transfer of Φm46.1: transfer and retransfer assays.

			Transductants			
				MIC (μg/ml)^a^									
Donor	Recipient	Transfer frequency	Genotype	ERY	TET
**Transfer assays^**b**^**
*S. pyogenes* m46	*S. pyogenes* 12RF	6.0 × 10^-4^	*mef*(A) *tet*(O)	16	64
*S. pyogenes* m46	*S. agalactiae* 1357RF	6.0 × 10^-7^	*mef*(A) *tet*(O)	16	64
*S. pyogenes* m46	*S. gordonii* 1435RF	4.3 × 10^-6^	*mef*(A) *tet*(O)	16	64
*S. pyogenes* m46	*S. suis* V36RF	2.3 × 10^-9^	*mef*(A) *tet*(O)	16	64
*S. pyogenes* m46	*S. pneumoniae* R6	NDT*^c^*			
*S. pyogenes* m46	*S. oralis* 1235RF	NDT			
*S. pyogenes* m46	*S. salivarius* 1555RF	NDT			
**Retransfer assays**
*S. pyogenes* 12RF-T^d^	*S. pyogenes* 12RF-SN	1.2 × 10^-5^	*mef*(A) *tet*(O)	16	64
*S. agalactiae* 1357RF-T	*S. pyogenes* 12RF-SN	1.6 × 10^-8^	*mef*(A) *tet*(O)	16	64
*S. gordonii* 1435RF-T	*S. pyogenes* 12RF-SN	2.8 × 10^-8^	*mef*(A) *tet*(O)	16	64
*S. suis* V36RF-T	*S. pyogenes* 12RF-SN	8.5 × 10^-4^	*mef*(A) *tet*(O)	16	64

*^a^ERY, erythromycin; TET, tetracycline.*

*^b^*Transfer data from *S. pyogenes* m46 to *S. pyogenes* 12RF are from previous experiments (Giovanetti et al., 2003).

*^c^NDT, no detectable transfer.*

*^d^The final T denotes a transductant obtained in the relevant transfer assay*.

Transductants exhibited a *mef* (A) *tet*(O) genotype and proved macrolide and tetracycline resistant, with MICs identical to those for the donors. The presence of a regular Φm46.1 was confirmed by PCR mapping in the transductants of all species. A transductant from each species was induced with mitomycin C: in all cases Φm46.1 was detected in culture supernatants in a DNAse-resistant form, such as a phage capsid.

### CHROMOSOMAL INTEGRATION SITE OF Φm46.1 IN DIFFERENT STREPTOCOCCAL SPECIES

**FIGURE 1:**
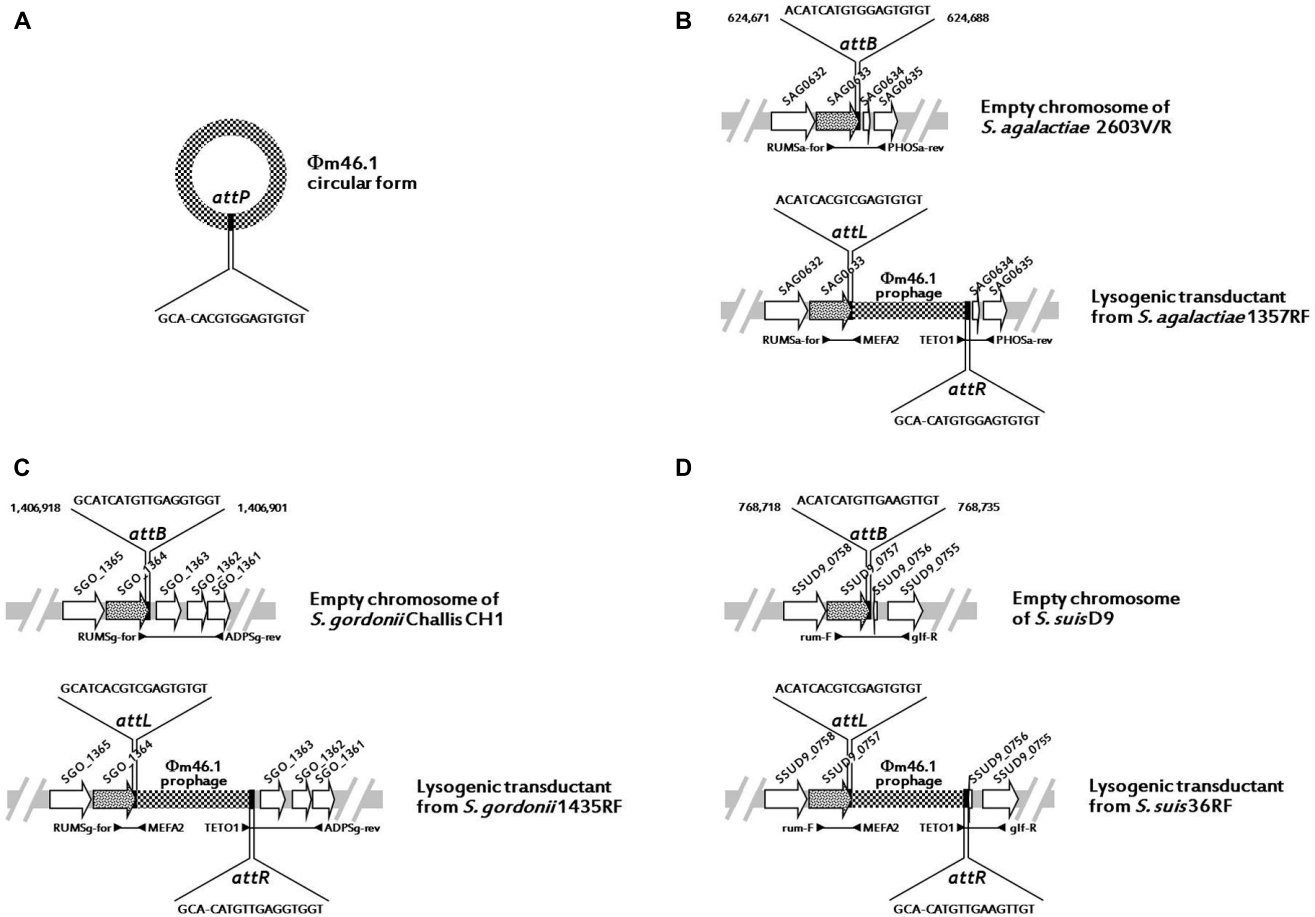
**Schematic representation (not drawn to scale) of Φm46.1 (checkered) in circular form with the *attP* sequence **(A)** and its integration into the chromosome of *S. agalactiae***(B)**, *S. gordonii***(C)**, and *S. suis***(D)**.** The chromosome is represented as a light gray bar. ORFs are represented as arrows pointing in the direction of transcription (the *rum*-like genes are spotted, the other genes are white). The top half of panels (**B–D**; the chromosome empty target) shows the relevant primer pair and the relevant *attB* sequence with the corresponding bases of the reference genome. The bottom half of panels (**B–D**; the lysogenic transductant) shows the relevant primer pairs for *attL* and *attR* and the respective sequences with the corresponding bases of the relevant transductant.

Since in *S. pyogenes* m46 Φm46.1 is integrated into the chromosome at the 3′ end of the *rum* gene (Brenciani et al., 2010), we first checked whether it had a similar integration site in the transductants obtained in the above experiments. This possibility was explored in one transductant of each species using two primer pairs, one for the left (*attL*) and one for the right (*attR*) junction. The primers of each pair that fell on the phage targeted *mef* (A) (left junction) or *tet*(O) (right junction), thanks to the close proximity of the two genes to the respective end of Φm46.1 (Brenciani et al., 2010). To design the primers that fell on the chromosome, homologs of the *rum* gene of *S. pyogenes* m46 in the relevant *Streptococcus* species (*S. agalactiae*, *S. gordonii*, and *S. suis*) were sought by BLASTN assays. A *rum*-like gene (DNA identity, around 70%) was detected in the genomes of such three species, and the one yielding the closest database match was considered. For *S. pyogenes*, the *rum* gene considered was Spy1197 from the genome of MGAS10750, 99% identical to the *rum* gene of *S. pyogenes* m46 and previously used as the reference *rum* gene when the Φm46.1 integration site was originally determined (Brenciani et al., 2010). The *rum*-like genes investigated are reported in **Table 3**, where the particular genes chosen to design the primers are also indicated. For *attL*, the primers to be paired to MEFA2, internal to *mef* (A), were designed from the respective *rum*-like gene in the portion upstream of the integration site; for *attR*, the primers to be paired to TETO1, internal to *tet*(O), were designed from open reading frames located downstream of the *rum*-like genes in the respective genomes (**Table 1**; **Figure 1**).

All PCR assays performed using these primer pairs yielded positive reactions, and all amplicons were sequenced and analyzed. The results indicated that in all transductants, irrespective of the species, the integration site of Φm46.1 corresponded to the one originally detected in *S. pyogenes* m46, i.e., at the 3′ end of the respective, species-specific *rum* gene. These data are schematically illustrated in **Figure 1**.

**Table 3:** *rum*-like genes and *attB* nucleotide sequences.

*rum*-like gene						
**From: *Streptococcus* species Strain (genome accession no.)**	**Designation^**a**^**	**DNA identity (%) to the *rum* gene of *S. pyogenes* m46**	**18-bp *attB* sequence^**b**^**

*S. pyogenes*						
MGAS10750^c^ (CP000262)	Spy1197^d^	99	GCATCACGTGGAGTGTGT
*S. agalactiae*						
2603V/R (AE009948)	SAG0633^d^	70	ACATCATGTGGAGTGTGT
*S. gordonii*						
Challis CH1 (CP000725)	SGO_1364^d^	67	GCATCATGTTGAGGTGGT
*S. suis*						
D9 (CP002641)	SSUD9_0757^d^	69	ACATCATGTTGAAGTTGT
*S. pneumoniae*						
ATCC 700669 (FM211187)	SPN23F09510	69	GCATCACGTGGAGTGCGT
*S. salivarius*						
57.I (CP002888)	Ssal_01495	71	GCACCATGTTGAGGCGGT
*S. oralis*						
Uo5 (FR720602)	SOR_1046	68	GCATCACGTTGAGTGTGT

*^a^Designations are as deposited in the database.*

*^b^Nucleotides in the *attB* sequences differing from the reference sequence of *S. pyogenes* MGAS10750 are underlined.*

*^c^*S. pyogenes* MGAS10750 is the strain whose *rum* gene was used as the reference when the Φm46.1 integration site was originally determined (Brenciani et al., 2010)*.

*^d^*rum*-like gene used for PCR primer design.*

### SEARCH FOR Φm46.1 CIRCULAR FORMS IN THE TRANSDUCTANTS AND CORE SITE ANALYSIS

PCR experiments using an appropriate pair of outward-directed primers (reverse primer targeting *orf1* and forward primer targeting *orf63* of Φm46.1; **Table 1**) were performed using one transductant per species. The circular form of Φm46.1 was consistently detected.

Sequence analysis of amplicons from the circular forms, the empty chromosomes, and the *attL* and *attR* regions of Φm46.1 from the *S. agalactiae*, *S. gordonii*, and *S. suis* transductants allowed identification of an 18-bp putative core site – i.e., the critical sequence where the site-specific recombination process presumably takes place – largely overlapping with the attachment sequence of Φm46.1 (*attP*) and the chromosomal attachment sequence (*attB*; **Table 3**). The *attB* site corresponded to bases 624,671 to 624,688 of the genome of *S. agalactiae* 2603V/R; to bases 1,406,918 to 1,406,901 of the genome of *S. gordonii* Challis *substr*. CH1; and to bases 768,718 to 768,735 of the genome of *S. suis* D9 (**Figure 1**).

### *rum*-LIKE GENES AND PUTATIVE CORE SITES OF Φm46.1 IN THE GENOMES OF THE SPECIES YIELDING NO TRANSDUCTANTS

*rum*-like genes ∼70% identical to the *rum* gene of *S. pyogenes* m46 were detected by BLASTN assays also in the genomes of the *Streptococcus* species yielding no transductants (*S. pneumoniae*, *S. oralis*, and *S. salivarius*). An 18-bp sequence largely overlapping with the above-mentioned *attB* sequences from *S. pyogenes*, *S. agalactiae*, *S. gordonii*, and *S. suis* was also detected in the *rum*-like genes from the genomes of these species (**Table 3**).

### FITNESS COST OF THE ACQUISITION OF Φm46.1

**FIGURE 2:**
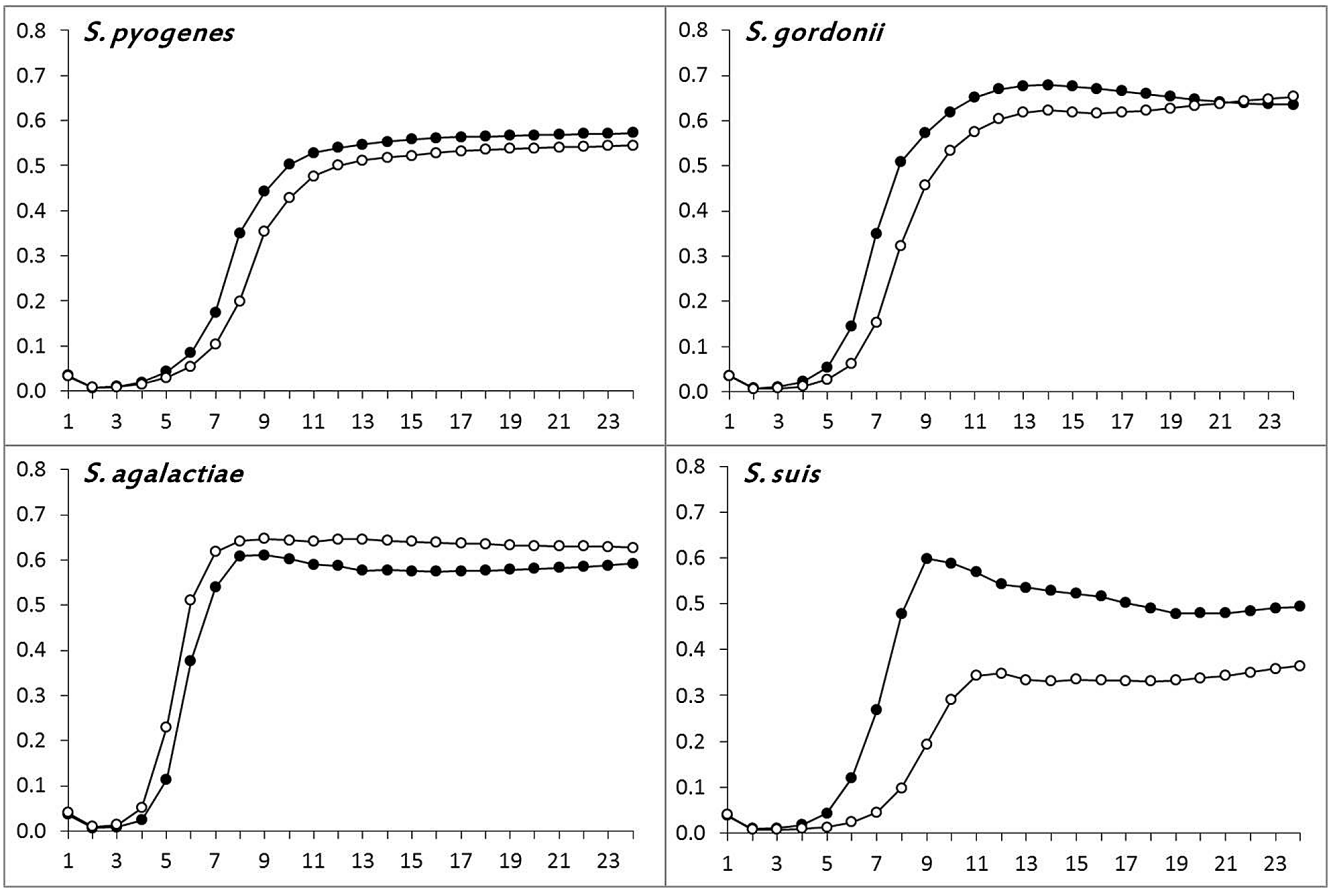
**Determination of bacterial fitness by growth assays.** Comparison of the growth rates of the recipient (°–°) and a randomly chosen transductant (∙–∙) of *S. pyogenes*, *S. agalactiae*, *S. gordonii*, and *S. suis*.

The *in vitro* growth curves of the recipient and the transductant of *S. pyogenes*, *S. agalactiae*, *S. gordonii*, and *S. suis* are shown in **Figure 2**. While the recipient and the transductant of three species (*S. pyogenes*, *S. agalactiae*, and *S. gordonii*) displayed similar growth rates, denoting a negligible impact of Φm46.1 acquisition on fitness, a distinctly greater fitness was observed in *S. suis*. In this species, the log phase started at 5 h in the transductant compared to 7 h in the recipient; moreover, the transductant displayed a greater growth rate compared to the recipient, with an earlier (at less than 9 h vs. 12 h) and higher (OD_690_, ∼0.60 vs. ∼0.35) peak.

Further insights into the behavior of *S. suis* appeared to be needed. Since a phage has recently been shown to alter fitness in *S. pneumoniae* by interfering with autolytic activity (DeBardeleben et al., 2014), and a novel autolysin-encoding gene (designated *atl*) has recently been described in *S. suis* (Ju et al., 2012), we investigated *atl* expression in the *S. suis* transductant and recipient. qRT-PCR experiments disclosed significant *atl* overexpression by the transductant compared to the recipient, the mRNA level in the former (300 copies) being at least 60-fold higher than in the latter (<5 copies).

## DISCUSSION

In bacteria bacteriophages are less common vehicles of antibiotic resistance genes than other mobile genetic elements such as plasmids or integrative and conjugative elements (ICEs). Φm46.1, the *S. pyogenes* phage carrying *mef* (A) and *tet*(O; Brenciani et al., 2010), is however, closely associated to a major erythromycin-resistant subpopulation of this species, which in Italy is predominant among M phenotype isolates (Spinaci et al., 2004; D’Ercole et al., 2005; Giovanetti et al., 2005). Φm46.1, common in *S. pyogenes* but unreported outside this species, is known to be transferable *in vitro* among *S. pyogenes* isolates (Giovanetti et al., 2003; Di Luca et al., 2010). In contrast, no information is available about its transferability to other *Streptococcus* species. The primary goal of our study was to explore this point. *In vitro* transfer assays showed that the recipients of some species (*S. agalactiae*, *S. gordonii*, and *S. suis*) yielded transductants harboring Φm46.1, whereas those of other species (*S. pneumoniae*, *S. oralis*, and *S. salivarius*) did not. In the transductants Φm46.1 retained its functional properties, including inducibility with mitomycin C, presence in the host cell both as a prophage and as free circular DNA, and transferability. In the species to which Φm46.1 was not transduced, failure to transfer did not seem to depend on the lack of the chromosomal integration site. Indeed, this site was consistently found to be at the 3′ end of a species-specific homolog of the *rum* gene, the chromosome integration site of Φm46.1 that was originally described in *S. pyogenes* (Brenciani et al., 2010); comparable *rum*-like genes were detected in the genomes of all *Streptococcus* species used as recipients, regardless of whether they yielded transductants. Failure of Φm46.1 to be transduced could perhaps reflect a flaw in an earlier step, e.g., during phage adsorption to host surface receptors or viral DNA injection.

A conserved *rum* gene is commonly found in streptococcal genomes, and its 3′ end is an integration hotspot for a vast array of genetic elements, typically carrying cargo genes encoding antibiotic resistances. In *S. pyogenes*, besides Φm46.1 [carrying *mef* (A) and *tet*(O)], such elements include ICE2096-RD.2 [*tet*(O)] (Beres and Musser, 2007); ICE*Sp*1108 [*erm*(TR)] (Brenciani et al., 2011); ICE*Sp*2905 [*erm*(TR) and *tet*(O)] (Brenciani et al., 2011); and ICE*Sp*2906 [*tet*(O)] (Giovanetti et al., 2012). Insertion into the same conserved *rum* location is also shared by *S. pyogenes* ICE6180-RD.1 (Beres and Musser, 2007) and the *S. agalactiae* prophage λSa04 (Brenciani et al., 2010), neither carrying resistance genes. In *S. suis*, it is important to mention ΦSsUD.1, a bacteriophage with a scaffold closely related to that of Φm46.1, which carries *tet*(W), a MAS (macrolide–aminoglycoside–streptothricin)-like fragment (Cochetti et al., 2005), and a *cadC/cadA* cadmium eﬄux cassette (Palmieri et al., 2011a). The same integration site is shared by a *S. suis* chimeric element (Hu et al., 2011; Palmieri et al., 2011b), constituted of an ICE and a phage, which carries *tet*(O) in tandem with *tet*(40), a *mef*(E)-containing mega-like structure (Gay and Stephens, 2001), and a MAS-like fragment. The *rum* 3′ region has very recently been reported to be the chromosomal integration site of genetic elements from other *Streptococcus* species: in particular of two related *vanG*-carrying elements conferring vancomycin resistance on *S. agalactiae* and *S. anginosus* (Srinivasan et al., 2014), previously unreported in these species. All these data actually suggested a strategy for routine localized screening of these insertions for the acquisition of new resistances (Srinivasan et al., 2014).

Of special interest was the marked difference between transfer and retransfer assays involving *S. suis*. While Φm46.1 was transferred from *S. pyogenes* to *S. suis* at a very low frequency (around 10^-9^), retransfer from *S. suis* to *S. pyogenes* occurred at a considerably greater (>10^4^ times) frequency. This may reflect the fact that *S. pyogenes* is the usual, natural host of Φm46.1, even though such a large difference between transfer and retransfer frequencies was not seen in the other species. On the other hand, the above-mentioned *S. suis* bacteriophage ΦSsUD.1 has proved to be transferable to *S. pyogenes*, but not to *S. suis* (Palmieri et al., 2011a).

Other intriguing findings, again concerning *S. suis* but not the transductants of the other species, were observed in fitness-related experiments. In *S. suis*, Φm46.1 acquisition was associated with a markedly greater growth rate and with overexpression of *atl*, an autolysin-encoding gene. These phenomena are experimentally very clear, but are not easy to elucidate; the reason why they occurred in *S. suis* but not in the other species is similarly difficult to explain. It is worth noting that the *atl*-encoded autolysin is believed to take part, besides cell autolysis, in separation of daughter cells, biofilm formation, fibronectin-binding activity, cell adhesion, and pathogenesis (Ju et al., 2012). In *S. pneumoniae*, the autolysin LytA has been shown to be activated after prophage induction and to contribute to efficient bacteriophage progeny release (Frias et al., 2009). Still in *S. pneumoniae*, autolysis-mediated fitness changes dependent on the presence of a prophage have recently been regarded as a new insight into how bacteria and prophages interact and affect bacterial fitness (DeBardeleben et al., 2014). The *atl* overexpression we observed in the *S. suis* transductant may suggests that the autolysin target, the cell wall, is more resistant to its enzymatic activity; however, unlike DeBardeleben et al. (2014) who observed increased penicillin resistance in a phage-harboring isolate, we found similar penicillin MICs for the *S. suis* transductant and recipient (data not shown). Moreover, it is worth noting that Φm46.1 contains a toxin–antitoxin system (Brenciani et al., 2010); such systems, possibly working as selfish entities favoring their own maintenance (Gerdes, 2000), may be involved in interactions with host regulatory networks and in fitness variations (Van Melderen and Saavedra De Bast, 2009).

A final consideration concerns the subpopulation naturally harboring Φm46.1, which in Italy is the majority of M phenotype erythromycin-resistant isolates of *S. pyogenes* (Spinaci et al., 2004; Giovanetti et al., 2005). The lack of a fitness cost associated with Φm46.1 acquisition by *pyogenes* may account for the wide circulation of such erythromycin- and tetracycline-coresistant organisms.

## Conflict of Interest Statement

The authors declare that the research was conducted in the absence of any commercial or financial relationships that could be construed as a potential conflict of interest.
